# Sodium doping in brookite TiO_2_ enhances its photocatalytic activity

**DOI:** 10.3762/bjnano.13.52

**Published:** 2022-07-05

**Authors:** Boxiang Zhuang, Honglong Shi, Honglei Zhang, Zeqian Zhang

**Affiliations:** 1 School of Science, Minzu University of China, 27 Zhong guancun South Avenue, Haidian District, Beijing, 100081, People's Republic of China

**Keywords:** brookite titanium dioxide, core–shell structure, photocatalytic activity, sodium doping, twins

## Abstract

We report in this work that sodium doping of brookite TiO_2_ effectively enhances its photocatalytic activity, which becomes three times higher than that of the quasi-spherical brookite TiO_2_. The results demonstrated that the sodium-doped brookite Na*_x_*Ti_1−_*_x_*O_2_ can be stable up to 500 °C. At 600°C, the sodium in the brookite precipitates in the form of Na_2_CO_3_, and above 700 °C, the brookite Na*_x_*Ti_1−_*_x_*O_2_ transforms into Na_2_Ti_6_O_13_ by a twinning process with the orientation relationship of [1−2−3]_Matrix_//[1−23]_Twins_ and (−2−10)_Matrix_//(1−1−1)_Twins_. The differences in the ionic radius and the electronegativity between Na and Ti destroy the local atomic arrangement of the brookite structure and produce microstructures such as the core–shell structure, local lattice distortion, interstitial atoms, and atomic vacancies, which are critical to its excellent photocatalytic activity.

## Introduction

Titanium dioxide (TiO_2_) has been extensively studied for many potential applications in the environmental and energy fields, such as treatment of polluted water [1–2], air purification [3–4], and water splitting [5–6]. In recent years, the interest in the brookite phase of TiO_2_ has been increased due to its importance for photocatalytic application. Ohtani et al. reported that extra-fine brookite TiO_2_ exhibited good photocatalytic activity for redox reactions in aqueous propan-2-ol and silver sulfate solution [7]. Kobayashi et al. suggested that the photoactivity of brookite nanoparticles exceeded four times that of P25 under visible light irradiation [8]. The degradation of methyl orange by brookite nanoflowers was much more efficient than that by anatase nanorods [9]. Moreover, the heterojunction of brookite TiO_2_ can enhance the photocatalytic activity (e.g., the rutile/brookite TiO_2_ heterojunction exhibited a synergetic effect, improving the photocatalytic activity for both hydrogen generation and organic dye degradation [10–11]). Similarly, the anatase/brookite heterojunction (38.2% brookite) also exhibited the highest degradation efficiency of cylindrospermopsin under UV–vis light [12].

The first synthesis of brookite TiO_2_ dates back to the late 1950s. But there are still many arguments on the structural and physical behaviors of brookite TiO_2_. Brookite was informally called "the least known TiO_2_" [13]. (1) The pure brookite TiO_2_ is difficult to be synthesized; in most cases, it mixes with anatase or rutile [14–15]. (2) The bandgap *E*_g_ is important for the photocatalytic behavior of brookite TiO_2_; however, the precise value of *E*_g_ is still unknown. The measured *E*_g_ value varies from 1.9 to 3.4 eV [16–17], and the theoretical value ranges from 1.8 to 3.3 eV [18]. (3) The performance of brookite mainly depends on the details of the preparation method and heat treatment used. For example, brookite TiO_2_ prepared from a Ti–EDTA complex exhibits a considerably higher activity for the degradation of NO than that synthesized from the titanate–glycolate complex [19]. The photodegradation of the orange II dye of brookite varies with the annealing temperature [20]. These differences in the photocatalytic activity of brookite were generally considered as factors such as the specific surface area, crystallite size, and crystallinity [9,21]. But whether the local atomic structure of brookite TiO_2_ changes with the preparing details is still unclear. The varied local structures of brookite can affect its electronic structure and hence its photocatalytic performance.

Here, we report that Na-doped brookite Na*_x_*Ti_1−_*_x_*O_2_ was synthesized by a hydrothermal reaction of tetrabutyl titanate, oxalic acid, and sodium hydroxide. It exhibits excellent photocatalytic activity in methylene blue solution (MB), which is about three times higher than that of the quasi-spherical brookite TiO_2_. The crystal structure of Na*_x_*Ti_1−_*_x_*O_2_ was determined by the Rietveld refinement method and verified by the atomic pair distribution function based on electron diffraction. High-resolution transmission electron microscopy results revealed the existence of a core–shell structure, lattice distortion, interstitial atoms, and atomic vacancies in Na*_x_*Ti_1−_*_x_*O_2_, which is critical for an excellent photocatalytic activity.

## Results and Discussion

### Enhanced photocatalytic activity

**Figure 1:**
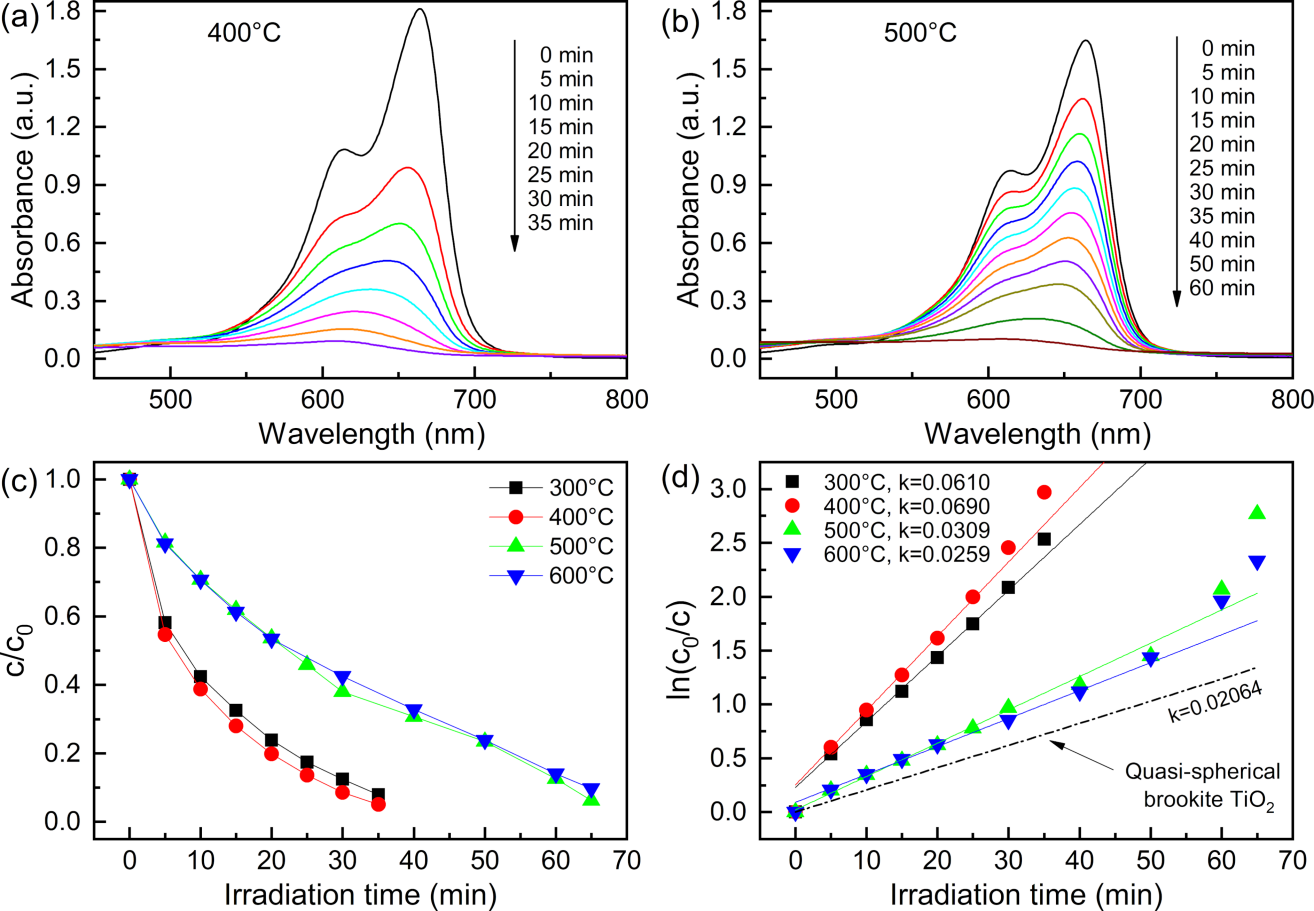
The UV absorption spectra of MB during photocatalytic reactions over samples calcinated at (a) 400 °C, (b) 500 °C. (c) The photodegradation of MB by samples calcinated at 300–600 °C. (d) Variations in ln(*c*/*c*_0_) as a function of the irradiation time, where the dotted line is the quasi-spherical brookite TiO_2_ reference (*k* = 0.0206).

The photocatalytic activity of the samples calcinated at 300–600 °C for 2 h was measured by the photodegradation of MB under UV light at room temperature. Figure 1a and Figure 1b display the dynamic changes in the absorption spectra of MB over two typical samples (400 and 500 °C). The MB concentration versus the irradiation time was determined from the characteristic absorption peak of MB (≈665 nm) acquired by a UV–vis spectrophotometer, as shown in Figure 1c. Samples calcinated at 300 and 400 °C exhibited an excellent photocatalytic activity, and more than 90% of MB was decolorized after UV light irradiation for 30 min. However, for the samples calcinated at 500 and 600 °C, it takes about 60 min to degrade 90% of MB. Moreover, the photodegradation rate of MB by the samples was quantitatively characterized based on pseudo first-order kinetics. Figure 1d displays the reaction rate constant (*k*) of samples calcinated at 300–600 °C to be 0.0610, 0.0690, 0.0309, and 0.0259 min^−1^, respectively. The degradation rate constant of the samples at 300 and 400 °C was enhanced about two times in comparison to that at 500 and 600 °C. By comparison, the reaction rate constant in this report was about three times higher than that of the quasi-spherical brookite TiO_2_ (*k* = 0.0206) [22]. The excellent photocatalytic activity can be correlated with the crystal structure, micromorphology, and chemical composition [23–25].

**Figure 2:**
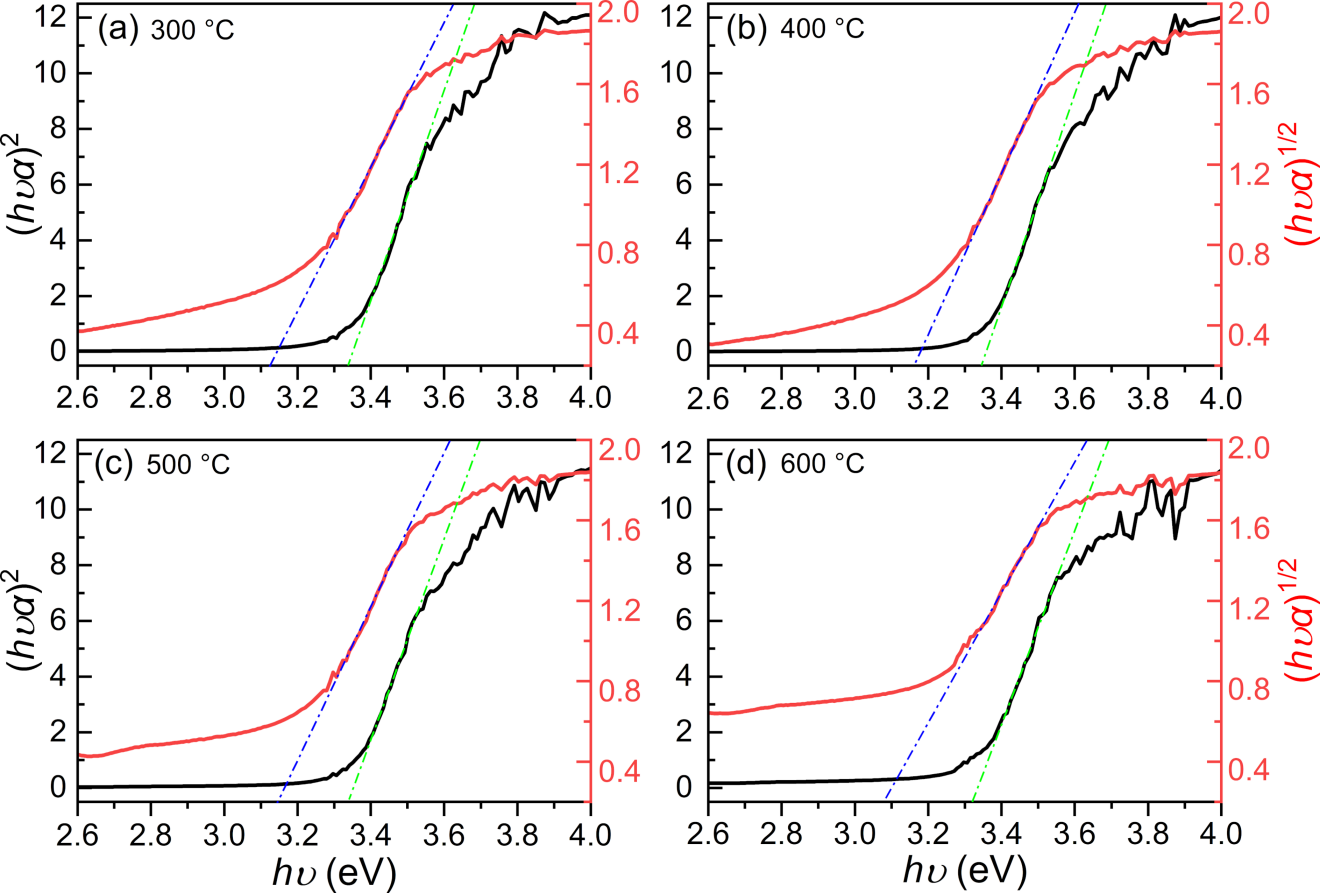
The bandgap values determined by the Tauc plot method from the diffuse reflectance spectra of samples calcinated at 300–600 °C. The black and red curves were calculated from the direct (*n* = 1/2) and indirect transition (*n* = 2), respectively.

The bandgap is useful to understand the photocatalytic behavior of a given material. The bandgap values were determined from the diffuse reflectance spectra using the Tauc plot method [26]:


[1] $${\left(hv\alpha \right)}^{\frac{1}{n}}=A\left(hv-E_{\text{g}}\right),$$

where *A* in Equation 1 is a proportional constant, α is the absorption coefficient, and *n* depends on the type of electron transition. The bandgap *E*_g_ is the *x*-axis interception when linear fitting the absorption edge of the Tauc plot. As both the direct and the indirect electron transition in the brookite were reported, two transitions were considered in this work. Figure 2 shows the Tauc plot derived from the diffuse reflectance spectra of samples calcinated at 300–600 °C, where the black curves were calculated from the direct transition (*n* = 1/2) and the red curves from the indirect transition (*n* = 2). The determined *E*_g_ for the direct transition ranged from 3.33–3.36 eV, while *E*_g_ for the indirect transition ranged from 3.02–3.11 eV (details are listed in Table 1). By comparison with the reported DFT calculation [18], the as-prepared brookite belongs to the direct electron transition. Despite the photocatalytic behavior of the samples at 300–400 °C being two times higher than that at 500–600 °C, there is no distinct difference in the bandgap values of these four samples.

**Table 1:** Listed are the direct/indirect bandgaps determined from diffuse reflectance spectra, the crystal size determined from the (211) diffraction peak, and the weight percentage of the brookite phase.

Temperature (°C)	*E*_g-direct_ (eV)	*E*_g-indirect_ (eV)	Crystal size (nm)	W% (Brookite)

300	3.35	3.07	41	67.4
400	3.36	3.11	39	82.1
500	3.35	3.09	39	82.8
600	3.33	3.02	43	83.3

### Structural phase diagram, chemical composition, and morphology

**Figure 3:**
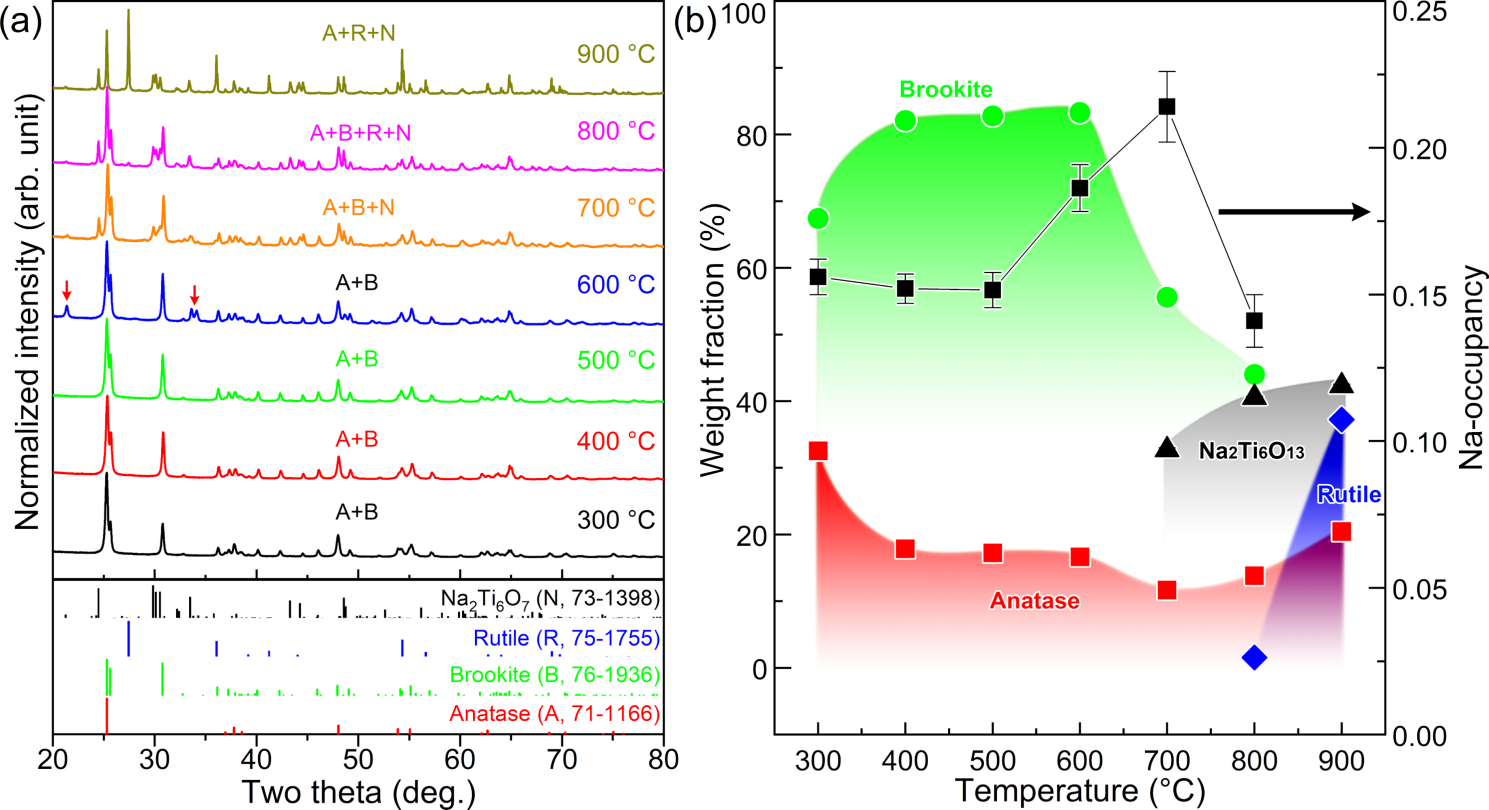
(a) The powder X-ray diffraction patterns of samples calcinated at 300–900 °C for 2 h, where the arrows indicate peaks of Na_2_CO_3_. (b) The temperature dependence of weight fractions and Na occupancy in the brookite phase.

The crystal structure of samples calcinated at 300–900 °C was characterized by powder X-ray diffraction (XRD), as shown in Figure 3a. The sample calcinated at 300 °C is a mixture of brookite (B) and anatase (A) with typical features: peaks at 25.6 and 30.8° come from the brookite phase alone; three peaks at 36.9, 37.8, and 38.5° belong to the anatase phase alone; and both the brookite and the anatase phases contribute to the most intense peak (at 25.3°). For the samples calcinated at 300–600 °C, the peak intensity of brookite at 25.6° is increased from 35% to 52%, suggesting a gradual increase of the brookite content. When the sample was calcinated above 700 and 800 °C, it was transformed into Na_2_Ti_6_O_13_ and rutile, respectively.

The crystal size is correlated to the specific surface area, which is considered to be one of the factors affecting photocatalytic performance. In this work, the Scherrer formula was employed to extract the crystal size from the (211) diffraction peak (details are listed in Table 1). Note that the extracted crystallite size is just the thickness of the (211) crystal plane, which is different from that observed via scanning electron microscopy (SEM) or transmission electron microscopy (TEM). Samples calcinated at 300–600 °C have a similar crystal size (ranging from 39–43 nm), suggesting that there is no obvious correlation between the crystal size and the photocatalytic activity of these samples.

Quantitative phase analysis of X-ray diffraction patterns provides the structure phase diagram, as shown in Figure 3b. It indicates that the weight fraction of the brookite phase can be up to 82% when samples were calcinated at 500 °C. Note that the sample at 600 °C precipitated a small amount of Na_2_(CO_3_) (PDF#86-0313), which suggests the existence of sodium in the samples. At 600 °C, the sodium in the samples shows thermal instability and can react with CO_2_ in the air and precipitate to form Na_2_(CO_3_). At 700 °C, brookite was directly transformed into Na_2_Ti_6_O_13_ (N) because the decrement of the brookite content (≈28%) was approximately equal to the increment of the Na_2_Ti_6_O_13_ (≈32%). Above 800 °C, both brookite and anatase transformed into the rutile phase (R), contributing to the rapid increment of the rutile fraction. Conversely, the Na_2_Ti_6_O_13_ phase increased only a little due to the finite content of sodium in the brookite phase.

**Figure 4:**
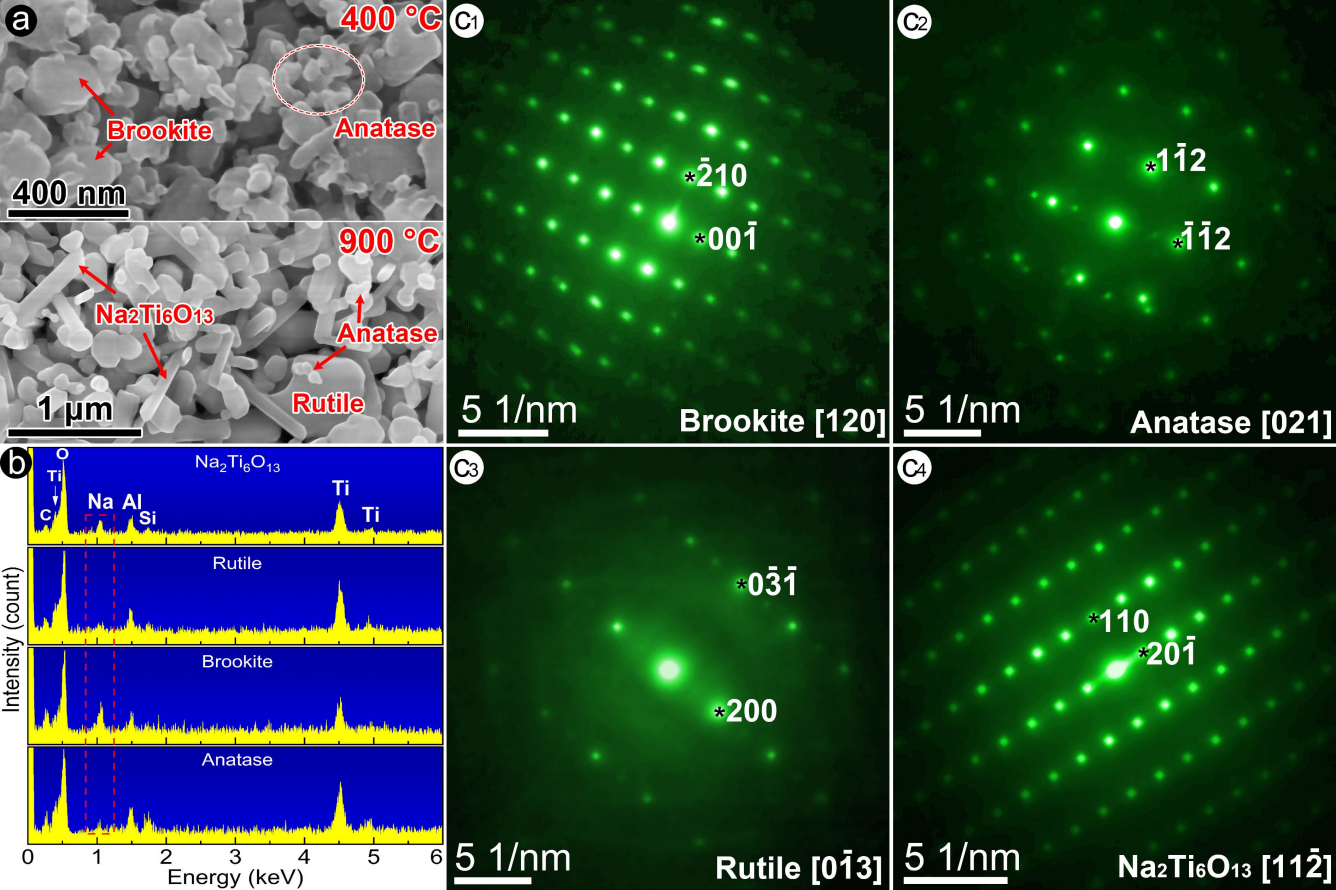
(a) Four typical SEM morphologies of samples calcinated at 400 and 900 °C: platy like brookite grains, fine anatase grains, block rutile grains, and Na_2_Ti_6_O_13_ rods. (b) The EDX spectrum acquired from four typical morphologies, and (c_1_–c_4_) electron diffraction patterns acquired from these four typical sample morphologies, where the corresponding HOLZ patterns are shown in Figure S1, Supporting Information File 1.

The X-ray diffraction experiment suggests the existence of sodium in the samples. Next, we verified in which structure sodium is present. Figure 4a shows four typical morphologies of products observed via SEM. Samples calcinated at 300–500 °C comprise platy brookite grains (≈100–300 nm in size) and fine anatase grains (≈50–70 nm in size). Samples calcinated at 700–900 °C comprise rod-like Na_2_Ti_6_O_13_ grains (≈100 nm in diameter and ≈600 nm in length), blocky rutile grains (≈300–600 nm in size), and fine anatase grains (≈50–80 nm in size).

(1) **Brookite—platy grains.** Energy-dispersive X-ray spectroscopy (EDX) results of platy grains (Figure 4b) indicate that Na can be well identified besides Ti and O (note that the characteristic peak of C comes from the carbon conducting resin, Al from the sample holder, and Si from the silicon wafer). The electron diffraction pattern (Figure 4c_1_ and Supporting Information File 1, Figure S1a) acquired from the platy crystallite can be indexed as the [120] zone axis of the brookite phase, of which the radius of the high-order Laue zone (HOLZ) ring was measured to be 23.58 nm^−1^ and the observed primitive cell volume was 254.7 Å^3^ as opposed to 258.9 Å^3^ for the standard brookite phase.

Note that the Na content in the brookite is affected by the process of centrifugation and washing of the samples. When the samples were centrifuged and washed three times (as described in the Experimental section), the Na content in the brookite significantly decreases. Details are displayed in Supporting Information File 1, Figure S3.

(2) **Anatase—fine grains.** Only Ti and O can be identified in the EDX spectra of fine grains. The electron diffraction pattern of the fine crystallite (Figure 4c_2_ and Supporting Information File 1, Figure S1b) can be determined as the [021] zone axis of the anatase phase with the HOLZ ring radius of 25.02 nm^−1^ and the primitive cell volume of 71 Å^3^ in comparison to 68.2 Å^3^ of the standard anatase structure.

(3) **Rutile—block grains.** Only Ti and O can be resolved in the EDX spectra of these blocks. The electron diffraction pattern (Figure 4c_3_ and Supporting Information File 1, Figure S1c) of the blocky crystallite can be indexed as the 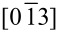 zone axis of the rutile phase with the HOLZ ring radius of 27.51 nm^−1^ and the primitive cell volume of 66 Å^3^ in comparison to 62.4 Å^3^ of the standard rutile phase.

(4) **Na_2_Ti_6_O_13_—rod-like crystals.** In addition to Ti and O, Na can be well identified in the EDX spectra of rod-like crystals. The electron diffraction pattern (Figure 4c_4_ and Supporting Information File 1, Figure S1d) from the rod-like crystallite can be identified as the 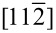 zone axis of the Na_2_Ti_6_O_13_ structure with the HOLZ ring radius of 26.86 nm^−1^ and the primitive-cell volume of 256 Å^3^ in comparison to 256.2 Å^3^ of the standard Na_2_Ti_6_O_13_ structure.

Here, we use the symbol Na*_x_*Ti_1−_*_x_*O_2_ (B) to indicate the as-synthesized brookite, TiO_2_ (B) for the conventional brookite, TiO_2_ (A) for the anatase phase, and TiO_2_ (R) for the rutile phase, respectively. The phase reaction occurring in the calcinating process can be described as follows:


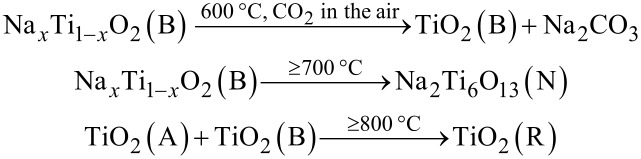


### The crystal structure of the brookite phase

**Figure 5:**
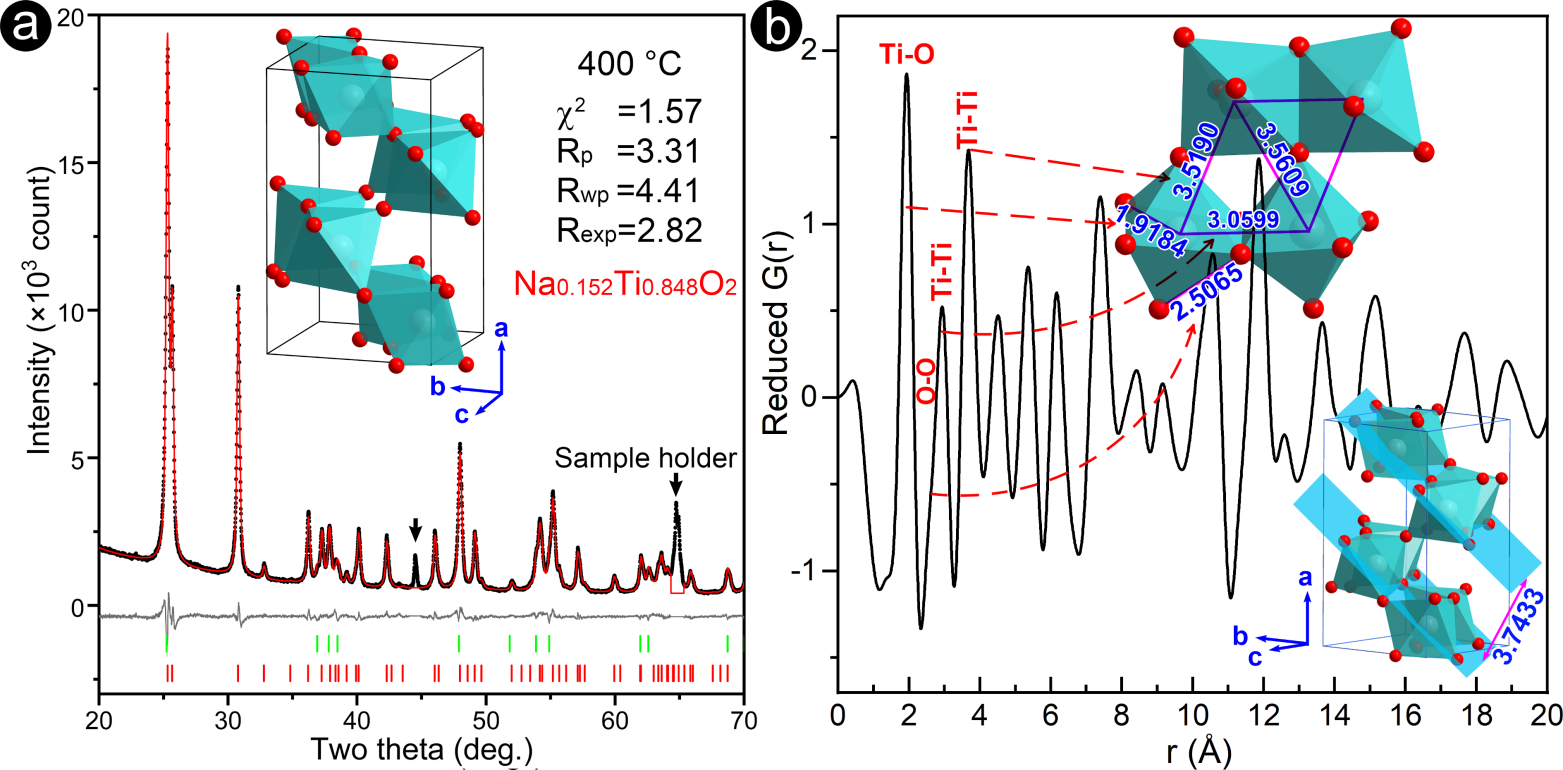
(a) A typical Rietveld refinement of the X-ray diffraction pattern of the sample calcinated at 400 °C. The positions of the Bragg reflections are indicated by vertical bars (red for Na_0.152_Ti_0.848_O_2_ and green for the anatase). The difference between the experimental (black dots) and the calculated (solid line) intensities is shown by the plot in the lower part of the pattern. (b) The reduced atomic pair distribution function *G*(*r*) extracted from the ring-like electron diffraction pattern, where the inset illustrates the local atomic structures of brookite.

The initial structure model of the as-prepared brookite phase is the orthorhombic brookite phase (ICSD#154605). However, the occupancy of the Ti atomic site is refined to be 0.8579(1), indicating that the Ti atomic site should be partially occupied by lighter Na atoms (due to the existence of sodium in the brookite phase). Figure 5a shows a typical Rietveld refinement pattern of the sample calcinated at 400 °C. The Rietveld *R* factors were *R*_p_ = 3.31, *R*_wp_ = 4.41, *R*_exp_ = 2.82, and χ^2^ = 1.57. In this sample, the weight fraction of the brookite phase is 82.1%, and the occupancy of sodium in the brookite structure is 0.152(5). Detailed atomic parameters of the structure Na_0.152_Ti_0.848_O_2_ and the anatase phase are listed in Table 2. For comparison, the pattern was also refined based on the standard brookite phase (ICSD#154605) [27]. The Rietveld *R* factors were *R*_p_ = 3.48, *R*_wp_ = 4.64, *R*_exp_ = 2.82, and χ^2^ = 1.65, a bit poorer than those of Na_0.152_Ti_0.848_O_2_. This indicates that the partial Na substitution of Ti atoms does not distinctly change the overall pattern of the brookite phase; instead, it slightly changes the intensity of some reflections.

**Table 2:** Atomic coordinates and occupancies of the crystal structures of the brookite Na_0.152_Ti_0.848_O_2_ and anatase TiO_2_ phases in the sample calcinated at 400 °C. The Rietveld *R* factors are *R*_p_ = 3.31, *R*_wp_ = 4.41, *R*_exp_ = 2.82, and χ^2^ = 1.57.

(1) Brookite phase Na_0.182_Ti_0.848_O_2_: *Pbca* (61), *a* = 9.18762(24) Å, *b* = 5.45909(13) Å, *c* = 5.14824(12) Å, α = β = γ = 90°, weight = 82.1(4)%.						

Atom	Wyck.	*x*	*y*	*z*	Occ.	U_iso_ (Å^2^)

Na	8c	0.12894(9)	0.09912(18)	0.86256(16)	0.152(5)	0.0049
Ti	8c	0.12894(9)	0.09912(18)	0.86256(16)	0.848(5)	0.0049
O1	8c	0.01302(27)	0.1529(4)	0.1860(4)	1	0.0077
O2	8c	0.23205(28)	0.1061(6)	0.5365(3)	1	0.0077

(2) Anatase phase TiO_2_: *I*4_1_/*amd* (141), *a* = *b* = 3.79571(13) Å, *c* = 9.5096(4) Å, α = β = γ = 90°, weight = 17.9(10)%.						

Atom	Wyck.	*x*	*y*	*z*	Occ.	U_iso_ (Å^2^)

Ti	4b	0.5	0.75	0.375	1	0.0049
O	8e	0.5	0.75	0.5837(4)	1	0.0077

Figure 3b shows the Na occupancy in the brookite Na*_x_*Ti_1−_*_x_*O_2_ as a function of temperature. The Na occupancy in the brookite maintained ≈0.15 when samples were calcinated at 300–500 °C, suggesting that the partial Na substitution of Ti atoms is beneficial to stabilizing the brookite structure. Above 600 °C, the Na occupancy in the brookite increased to ≈0.2, causing thermal instability of the brookite structure (i.e., precipitating Na_2_CO_3_ or transforming into Na_2_Ti_6_O_13_). Some reports [9,28–30] indicated that the content of the brookite phase increases with the supplement of sodium sources but it is still difficult to synthesize pure brookite, meaning that there must exist an upper limit of sodium occupying the brookite. Considering that the brookite Na*_x_*Ti_1−_*_x_*O_2_ can directly transform into the Na_2_Ti_6_O_13_ structure, the content of sodium in the brookite Na*_x_*Ti_1−_*_x_*O_2_ can be estimated when Na_2_Ti_6_O_13_ is written as Na_0.25_Ti_0.75_O_1.625_ (i.e., *x*_max_ = 0.25), which is consistent with I. Keesmann's phase diagram [30].

To verify the refined crystal structure, we extracted the atomic pair distribution function (PDF) from the ring-like electron diffraction pattern, as shown in Figure 5b. The typical features of the PDF curve are: (1) the first peak at ≈1.9 Å is the shortest Ti/Na–O bond and the measured coordination number approaches six, indicating the existence of Ti/Na–O_6_ octahedra in the specimen. (2) The neighboring O–O bonds in octahedra contribute to a PDF shoulder at ≈2.5 Å. (3) The third peak at ≈3 Å corresponds to the nearest neighboring Ti–Ti bonds, indicating that two neighboring octahedra are packed in an edge-sharing way. (4) The fourth peak at ≈3.5 Å is a broadened peak, indicating that there is a small difference between the two next nearest neighboring Ti–Ti bonds. So that, three neighboring Ti/Na atoms compose an approximate isosceles triangle (3.0599 Å for the base, 3.5190 and 3.5609 Å for the sides, as illustrated in Figure 5b), generating an octahedron layer with the lattice-plane indices 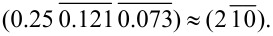 Two octahedron layers spacing ≈3.7433 Å share corners to construct the brookite structure, as depicted in the inset of Figure 5b.

### The core–shell structure, defects, and twins in the brookite

**Figure 6:**
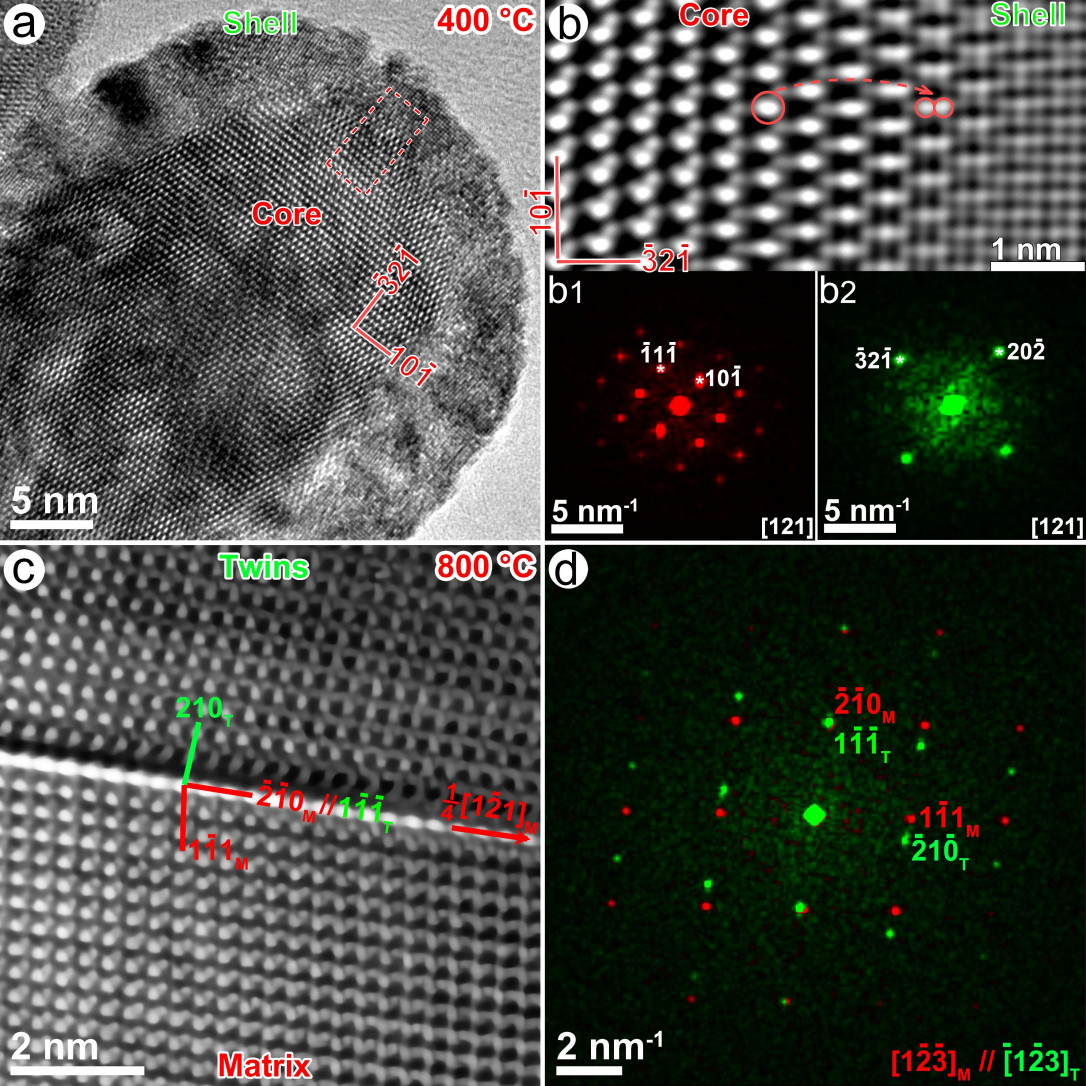
(a) An HRTEM image exhibits the core–shell structure in a brookite crystallite calcinated at 400 °C. (b) The magnified HRTEM image in the dashed box shows an atom-splitting effect. (b1–b2) The Fourier transformation diffractogram of the core and the shell. (c) The HRTEM image of a twinning boundary in a brookite crystallite calcinated at 800 °C, (d) the corresponding Fourier transformation diagram of the matrix (red) and the twins (green).

Considering the differences in the ionic radius and the electronegativity between Na and Ti, the Na doping in the Ti site will destroy the local atomic arrangement of the brookite phase and produce some microstructures. Figure 6a displays a typical high-resolution transmission electron microscopy (HRTEM) image of the sample calcinated at 400 °C, oriented at the [121]_Brookite_ zone axis. The crystallite exhibits a core–shell-like structure (i.e., a shell with a non-uniform thickness of ≈1–6 nm which grows around a core of ≈25 nm in width). Figure 6b shows a magnified image of the core–shell boundary (the dashed box in Figure 6a). The shell is a squared Ti–O structure (≈1.9 Å between the neighboring atomic columns), formed by splitting Ti atoms along the 
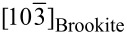
 direction. In addition, the Na doping in the Ti site of the brookite structure can destroy the local atomic arrangement, as shown in Supporting Information File 1, Figure S4. In the sample at 400 °C, the ordered atomic arrangement is only confined within the range of 1–3 nm; here, we call it the nanodomain. One nanodomain can develop into the neighboring domain by lattice distortion (Supporting Information File 1, Figure S4a_1_), interstitial atom, and atomic vacancy (Supporting Information File 1, Figure S4a_2_).

However, these microstructures including the core–shell structure, the nanodomain, interstitial atoms, atomic vacancies, and complex defects can be frequently observed in the samples calcinated at 300 and 400 °C but rarely for other samples calcinated at higher temperatures. Since the higher calcinating temperature can improve the atomic migration rate, atoms at higher potential energy sites will migrate to the stable site and finally develop into a perfect crystallite. For example, the atomic arrangement within the brookite crystallite in Figure 6c becomes more ordered than that calcinated at a lower temperature, and the size of the brookite crystallite reaches ≈100 nm beside a twin boundary.

The twining structure is formed by atoms in the 
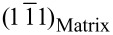
 plane which successively displaced 1/4 units along the 
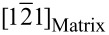
 direction. The mismatch of the twinning structure is ≈1.35% because the *d*-spacing of the 
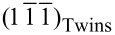
 plane is approximately equal to the 
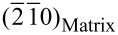
 plane whose interplanar spacing values are 3.5092 and 3.4619 Å, respectively. Thus, the orientation relationship between the matrix and twins is 
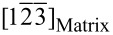
//
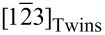
 and 
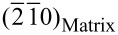
//
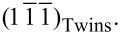
 The twinning effect in the brookite structure may be the major driven force of the phase transformation from the brookite Na*_x_*Ti_1−_*_x_*O_2_ to Na_2_Ti_6_O_13_. Such transformation twins are commonly observed in BaTiO_3_ [31] and CaTiO_3_ [32].

## Conclusion

A sodium-doped brookite Na*_x_*Ti_1−_*_x_*O_2_ was synthesized in NaOH solution. It exhibits an excellent photocatalytic activity, three times higher than that of the quasi-spherical brookite TiO_2_. Results of X-ray diffraction indicate that sodium doping can stabilize the brookite Na*_x_*Ti_1−_*_x_*O_2_ up to 500 °C. At 600 °C, sodium in the brookite precipitates in the form of Na_2_CO_3_ and above 700 °C, the brookite Na*_x_*Ti_1−_*_x_*O_2_ directly transforms into the Na_2_Ti_6_O_13_ phase via a twining process. The twinning structure is formed by successively displacing atoms in the 
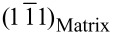
 plane along the 1/4
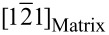
 direction, and the twinning relationship is 
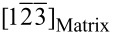
//
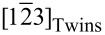
 and 
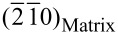
//
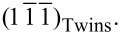
 The photocatalytic behavior of the Na-doped brookite has no obvious correlation with the Na content. However, the differences in the ionic radius and the electronegativity between Na and Ti can destroy the local atomic arrangement of the brookite phase and produce many microstructures such as the core–shell structure, the lattice distortion, interstitial atoms, and atomic vacancies. These interstitial atoms, surface vacancies, and the incomplete structure are considered as photocatalytic active sites that can enhance the photocatalytic activity [33–34]. However, the high temperature can improve the atomic migration rate which makes the atomic arrangement more ordered. In other words, the high temperature relaxes the lattice distortion and smoothen the incomplete structure while weakening the photocatalytic behavior.

This report shows that sodium can be doped in brookite TiO_2_ by the conventional hydrothermal method, meaning that other alkali anions are also expected to form the brookite A*_x_*Ti_1−_*_x_*O_2_ (A = Na, K, Ca). We suggest giving more attention to the local atomic structure and the chemical composition of brookite TiO_2_ that is hydrothermally synthesized in alkaline solution. Additionally, the squared Ti–O structure in the core–shell structure is also expected for a high performance of the photocatalytic activity.

## Experimental

### Materials

*N*,*N*-Dimethylformamide (AR, ≥99.5%), oxalic acid dihydrate (AR, ≥99.5%), and methylene blue (≥98.0%) were purchased from Sinopharm Chemical Reagent Co. Tetrabutyl titanate (TBOT, ≥99.0%) and sodium hydroxide (≥98%) were purchased from Aladdin Biochemical Technology Co. Absolute ethanol (AR, ≥99.7%) was purchased from Beijing Innochem. Science & Technology Co. All reagents and solvents were used without further purification.

### Hydrothermal synthesis of the Na-doping brookite

In a typical synthesis procedure, 1.27 g of oxalic acid dihydrate was dissolved into 50 mL of *N*,*N*-dimethylformamide (DMF) solvent to obtain a colorless and transparent solution under vigorous stirring. Then 1.5 mL of TBOT was slowly dropped into the solution to form a transparent yellow sol by vigorously stirring for a full reaction between TBOT and oxalic acid. The pH value of the sol was adjusted to 10 by using 2 mol/L of NaOH solution. After stirring for another 1 h, the sol was transferred into a 100 mL Teflon-lined autoclave and heated at 180 °C for 8 h. The white precipitate was collected by centrifugation (8000 rpm, 10 min), washed to a neutral pH with distilled water and absolute ethanol, and then dried at 80 °C. To inspect the effect of the calcinating temperature on the structure of the titanium dioxide, 7 g of the precipitate from different batches was uniformly mixed and divided into seven parts, which were then calcinated at 300–900 °C for 2 h to obtain the products.

### Photocatalytic activity measurement

The photocatalytic activity of the products was examined by photodegrading the methylene blue (MB) solution at room temperature under UV light generated by a PLS-SXE300 Xenon lamp with a 365 nm band filter. Following the method reported by Wang et al. [22], 15 mg of the catalyst was dispersed into 75 mL of the MB solution (10 mg/L). Before UV light irradiation, the suspension was magnetically stirred for 30 min in the dark to equilibrate the adsorption and desorption of MB on the surface of the crystallites. After switching on the UV light, samples were periodically collected and centrifuged. The concentration of the MB solution was analyzed based on the characteristic absorption peak of the MB (≈665 nm) acquired by a UV–vis spectrophotometer.

The diffuse reflectance spectra were measured in the wavelength range of 300–800 nm using a UV–vis spectrophotometer. The bandgap was determined by linear fitting the absorption edge of the Tauc plot of the absorption spectra.

### Structure characterization

To inspect the structure evolution of the titanium dioxide with the calcinating temperature, the as-prepared products were examined by an X-ray diffractometer (Bruker D8 Advance) using Cu Kα radiation (λ = 1.5406 Å). The Rietveld refinement of patterns was performed in the GSAS-II software [35].

The morphology and the chemical composition of the products were characterized by a field-emission scanning electron microscope (Hitachi S-4800) equipped with an energy-dispersive X-ray spectroscope working at 10 kV and 10 μA. Samples were dispersed into ethyl alcohol and then sprayed on a silicon wafer pasted on an aluminum sample holder by a carbon conducting resin. The EDX spectra were acquired from individual morphologies or grains in the samples. Electron diffraction and high-resolution imaging experiments were carried out on a high-resolution transmission electron microscope (JEOL JEM-2100) operating at 200 kV. To identify the crystal structure of the nanocrystallite, we employed the pattern indexing method in combination with the high-order Laue diffraction ring which can provide the primitive-cell volume, an important crystallographic parameter. Packages of electron diffraction tools [36] and ePDF tools [37] were used to perform pattern indexing and to extract the atomic pair distribution function (PDF).

## Supporting Information

File 1 Additional figures.
