# Supplementary material for: Sodium doping in brookite TiO2 enhances its photocatalytic activity
Source: Beilstein J Nanotechnol. 2022 Jul 5;13:599–609. doi: 10.3762/bjnano.13.52 (PMC9273989; doi:10.3762/bjnano.13.52)
Supplement: File 1 — Additional figures. [file Beilstein_J_Nanotechnol-13-599-s001.pdf]

BEILSTEIN JOURNAL OF NANOTECHNOLOGY

Supporting Information

for

Sodium doping in brookite TiO_2 enhances its photocatalytic activity

Boxiang Zhuang, Honglong Shi, Honglei Zhang and Zeqian Zhang

Beilstein J. Nanotechnol. **2022,** *13,* 599–609. doi:10.3762/bjnano.13.52

Additional figures

License and Terms: This is a supporting information file under the terms of the Creative Commons Attribution License (https://creativecommons.org/licenses/by/4.0). Please note that the reuse, redistribution and reproduction in particular requires that the author(s) and source are credited and that individual graphics may be subject to special legal provisions.
The license is subject to the *Beilstein Journal of Nanotechnology* terms and conditions: (https://www.beilstein-journals.org/bjnano/terms)

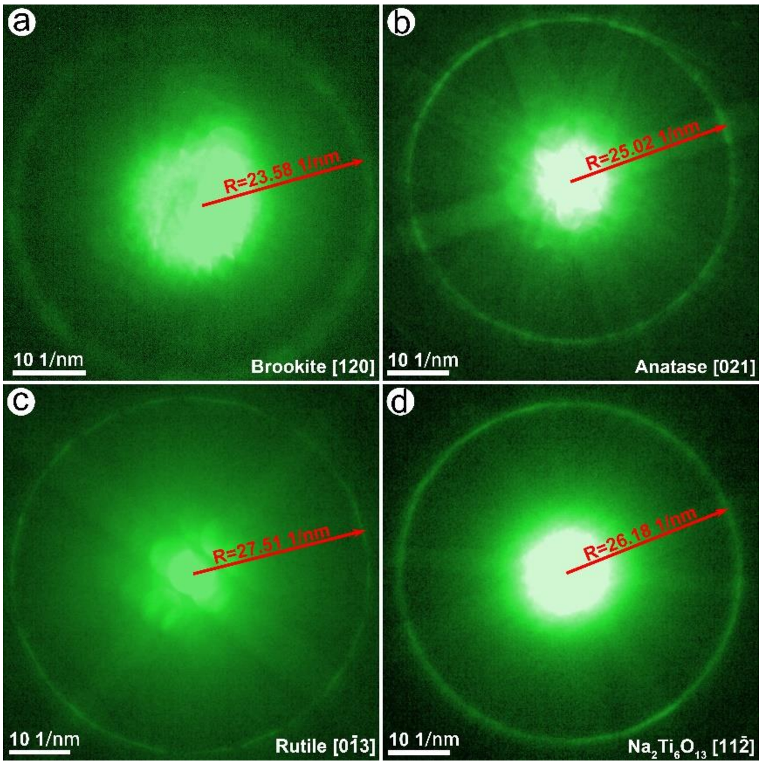

Figure S1: High-order Laue zone patterns of (a) brookite, (b) anatase, (c) rutile, and (d) $Na_2Ti_6O_{13}$, corresponding to the SAED patterns in Figure 4c$_1$–4c$_4$.

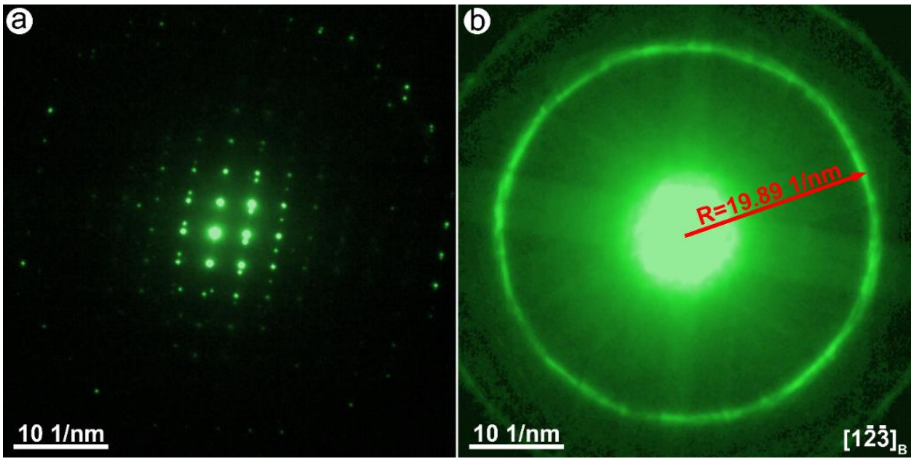

Figure S2: The SAED and high-order Laue zone patterns of the brookite crystallite calcinated at 800 °C, corresponding to the HRTEM image and the Fourier diffractogram in Figure 6c–6d.

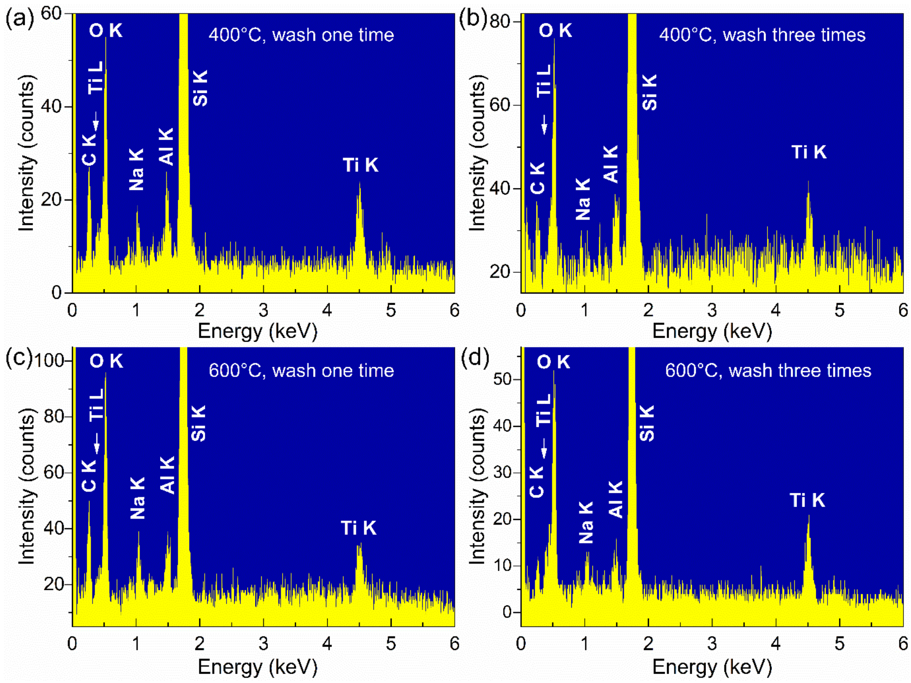

Figure S3: Comparison of the Na content of the as-obtained brookite when the hydrothermal reaction precipitates were centrifuged and washed (as described in the Experimental Section) one and three times, and then calcinated at 400 and 600 °C, respectively. It indicates that the Na content of the brookite clearly decreases after being washed three times.

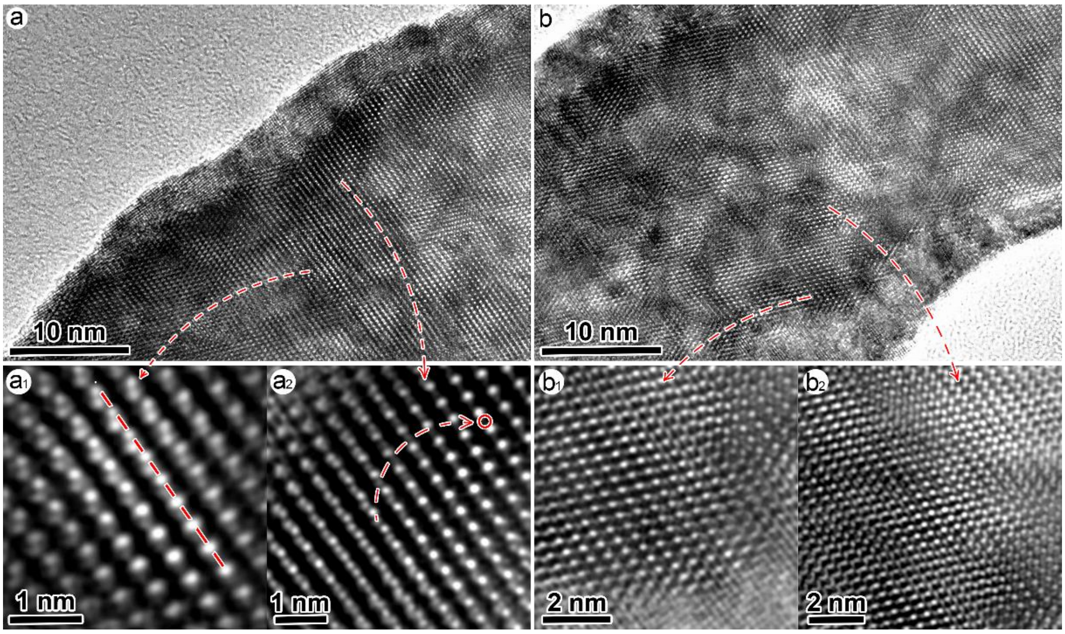

Figure S4: The HRTEM images of the brookite crystallite calcinated at 400 °C. There is a large number of (a_1) local lattice distortions, (a_2) interstitial atoms and atomic vacancies, and (b_1–b_2) complex defects.
